# Trends in Celecoxib Prescribing: A Single Institution 16-Month Review

**DOI:** 10.3390/jcm14082823

**Published:** 2025-04-19

**Authors:** Ivo H. Cerda, Helen Jung, Maria C. Guerrero, Rodrigo Diez Tafur, Robert Jason Yong, Christopher L. Robinson, Jamal J. Hasoon

**Affiliations:** 1 Harvard Medical School, Boston, MA 02115, USA; ivocerda@hms.harvard.edu; 2 Albert Einstein College of Medicine, Bronx, NY 10461, USA; 3 Department of Physical Medicine & Rehabilitation, Larking Community Hospital, South Miami, FL 33143, USA; 4 Pain Management Unit, Clínica Angloamericana, San Isidro 15073, Peru; 5 Centro MDRS: Sports, Spine & Pain Centers, Miraflores 15073, Peru; 6 Department of Anesthesiology, Perioperative, and Pain Medicine, Harvard Medical School, Boston, MA 02115, USA; ryong@bwh.harvard.edu (R.J.Y.); christopherrobinsonmdphd@outlook.com (C.L.R.); 7 Department of Anesthesia, Critical Care, and Pain Medicine, UTHealth, McGovern Medical School, Houston, TX 77030, USA

**Keywords:** celecoxib, COX-2 inhibitors, nonsteroidal anti-inflammatory drugs, chronic pain, pain management

## Abstract

**Background/Objectives:** Celecoxib, a COX-2 selective nonsteroidal anti-inflammatory drug (NSAID), is widely prescribed for pain management due to its efficacy and improved gastrointestinal safety profile compared to traditional NSAIDs. Understanding prescription trends and their comparison to other NSAIDs provides valuable insight into prescribing behaviors in clinical settings. **Methods:** This retrospective study analyzed celecoxib prescriptions written by three pain management physicians in a single institution over a 16-month period from 1 January 2023 to 30 April 2024. Prescription data were collected and grouped into four 4-month intervals to assess temporal trends. Additionally, we compared celecoxib prescriptions to other commonly prescribed NSAIDs, including ibuprofen, meloxicam, naproxen, and diclofenac. **Results:** A total of 143 celecoxib prescriptions were identified during the study period, with a steady increase observed across consecutive intervals: 8 prescriptions from January–April 2023, 22 from May–August 2023, 46 from September–December 2023, and 67 from January–April 2024. In comparison, a total of 165 prescriptions were written for other NSAIDs over the same period, with 26 prescriptions from January–April 2023, 41 from May–August 2023, 45 from September–December 2023, and 53 from January–April 2024. While prescriptions for both celecoxib and other NSAIDs increased over time, the rate of celecoxib prescriptions showed a steeper rise. **Conclusions:** The findings demonstrate a notable increase in celecoxib prescriptions in this pain management clinic, outpacing the growth of other NSAIDs. This trend may reflect increasing provider preference for COX-2 selective inhibitors due to their favorable safety profile and efficacy. Further research is warranted to explore the underlying factors driving these prescribing patterns.

## 1. Introduction

Chronic pain represents a significant public health concern, affecting approximately 1 in 5 adults in the United States [1]. Defined as pain persisting or recurring for at least three months [2], chronic pain imposes a profound physical, emotional, and economic burden on patients and healthcare systems [3,4,5]. Individuals experiencing chronic pain report poorer quality of life [6], difficulty in performing basic daily activities [7], and increased symptoms of depression and anxiety [8] when compared to those without chronic pain.

The economic impact of chronic pain is substantial, with estimates from two decades ago indicating that it costs the U.S. healthcare system between USD 560 and USD 635 billion, which encompasses healthcare expenses, disability-related costs, and productivity losses [9], and have only continued to rise over time [3,10]. Given these far-reaching consequences, the development of effective and sustainable pain management strategies has become a critical priority for clinicians and researchers. However, the complex and multifactorial nature of chronic pain, driven by diverse and often poorly understood pathophysiological mechanisms, necessitates a multimodal approach to optimize outcomes [11,12].

Nonsteroidal anti-inflammatory drugs (NSAIDs) have long been a cornerstone of pain management due to their ability to reduce inflammation and alleviate pain. Traditional NSAIDs, such as ibuprofen, naproxen, and diclofenac, are non-selective inhibitors of both cyclooxygenase-1 (COX-1) and cyclooxygenase-2 (COX-2) [13]. While COX-2 inhibition is thought to mediate their therapeutic effects, simultaneous inhibition of COX-1 is thought to disrupt gastrointestinal mucosal protection and platelet function, increasing the risk of ulcers and bleeding [14]. These adverse effects can limit their long-term use, particularly in patients with comorbid conditions. As a result, since before the turn of the century, selective COX-2 inhibitors, such as celecoxib, have garnered increasing interest as alternatives to non-selective NSAIDs. Accumulating evidence now suggests that these agents provide comparable analgesic efficacy while offering an improved safety profile, particularly with respect to COX-1-mediated gastrointestinal toxicity [15]. These agents have emerged as a valuable item in the clinician’s toolbox, and as a particularly good option for patients requiring long-term pain relief with a lower risk of gastrointestinal complications.

Celecoxib exerts its analgesic and anti-inflammatory effects by inhibiting COX-2, thereby decreasing prostaglandin synthesis, while sparing COX-1 at therapeutic concentrations [16]. Its clinical efficacy has been demonstrated in numerous clinical trials and real-world studies. Randomized control trials have shown its efficacy in chronic nonspecific low back pain, where it outperforms acetaminophen [17], in osteoarthritis (OA), demonstrating superiority to a placebo [18,19,20] and diclofenac [21], and in juvenile rheumatoid arthritis (JRA), where it has shown similar efficacy to naproxen [22], among other conditions.

Celecoxib was the first selective COX-2 inhibitor introduced into clinical practice and has since become one of the most widely utilized NSAIDs for pain management. As a non-opioid analgesic, it offers a viable alternative for patients requiring sustained analgesia, aligning with broader efforts to reduce opioid reliance in clinical practice. Its role in multimodal pain management strategies is supported by current clinical guidelines [23,24,25,26], including those from the World Health Organization (WHO), which emphasizes non-opioid therapies as first-line treatments for chronic pain [27].

Despite its growing role, the utilization of celecoxib in specialized pain management settings remains underexplored. Understanding prescribing patterns within these clinics can provide valuable insights into its role in addressing the needs of complex patient populations. The clinic in which this study was conducted is part of a larger academic healthcare institution and primarily manages patients with chronic spinal pain, osteoarthritis, neuropathic pain, and postoperative pain. On average, the clinic sees approximately 300–350 patients per month for clinic visits, overseen by three board-certified pain management physicians. Celecoxib was selected as the focus of this study because of its increasing prominence as a preferred COX-2 inhibitor in pain management, known for its efficacy and safety profile. This study aims to bridge this gap by analyzing celecoxib prescribing trends over a 16-month period in a single pain management clinic. By identifying these trends, the study seeks to contribute to the growing body of knowledge on the integration of COX-2 inhibitors in chronic pain management.

## 2. Methods

### 2.1. Study Design

This study was designed as a retrospective analysis of NSAID prescribing trends within a single pain management clinic. The clinic is part of a large academic medical center located in Houston, Texas. The primary focus was on identifying and quantifying all prescriptions for celecoxib, with a comparative analysis of other commonly prescribed NSAIDs, including ibuprofen, meloxicam, naproxen, and diclofenac, issued by three board-certified pain management physicians over a 16-month period. The timeframe for this study was from 1 January 2023 to 30 April 2024. By examining prescribing patterns over this extended duration, the study sought to capture temporal variations and provide insight into shifts in prescribing behaviors in response to clinical needs, guidelines, or institutional practices.

This study did not involve the collection of patient-specific data, as it solely captured the total number of prescriptions issued within the department. No patient records were accessed, and all data were anonymized. Therefore, Institutional Review Board (IRB) approval was not required, as the study did not involve human subject research. All ethical standards were followed in accordance with institutional policies on retrospective data collection and analysis. Artificial intelligence tools were not used to generate any of the text for the manuscript. ChatGPT4.0 was used to assist in the creation of the figures.

### 2.2. Data Collection

Prescription data were systematically reviewed and extracted from the clinic’s electronic medical records. Records were filtered to identify prescriptions for celecoxib as well as other commonly prescribed NSAIDs, including ibuprofen, meloxicam, naproxen, and diclofenac, ensuring accuracy in quantifying their use over the study period. To facilitate meaningful analysis, the data were grouped into four 4-month intervals: January–April 2023, May–August 2023, September–December 2023, and January–April 2024.

Each interval represented a distinct phase of prescribing behavior, allowing for the identification of fluctuations and incremental changes that might otherwise go unnoticed in a broader time frame. Both celecoxib and other NSAID prescriptions were analyzed separately to assess comparative prescribing trends. To maintain data integrity, only unique prescriptions during each period were included, with refills excluded to avoid duplication. This approach ensured a clear and accurate representation of NSAID prescribing trends over time.

## 3. Results

Over the 16-month study period, a total of 143 prescriptions for celecoxib were identified, showing a consistent upward trend over time. The number of celecoxib prescriptions issued in each 4-month interval was as follows: 8 prescriptions from January–April 2023, 22 from May–August 2023, 46 from September–December 2023, and 67 from January–April 2024.

In comparison, a total of 165 prescriptions for other NSAIDs (ibuprofen, meloxicam, naproxen, and diclofenac) were identified over the same period. The distribution of these prescriptions was as follows: 26 prescriptions from January–April 2023, 41 from May–August 2023, 45 from September–December 2023, and 53 from January–April 2024.

While prescriptions for both celecoxib and other NSAIDs increased over time, celecoxib prescriptions exhibited a steeper rate of growth, surpassing the total number of other NSAID prescriptions in the later months of the study period. This trend suggests an increasing preference for celecoxib within the pain management clinic.

**Figure 1:**
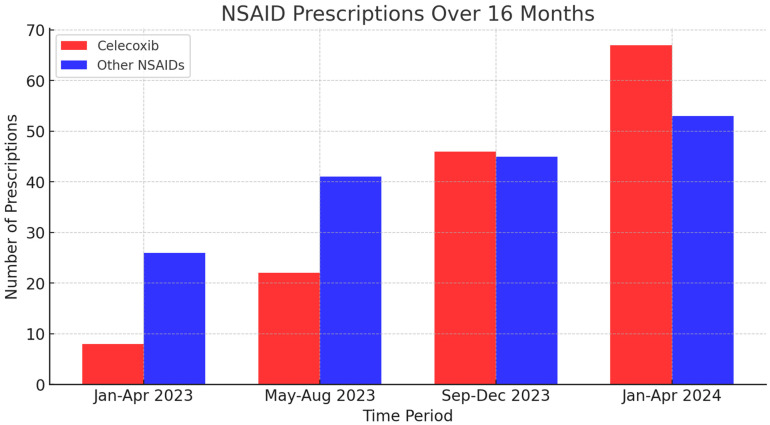
This bar graph illustrates the total number of celecoxib (red) and other NSAIDs (blue) prescriptions issued in a pain management clinic over four consecutive 4-month intervals from January 2023 to April 2024. The data demonstrate a steady increase in the number of prescriptions for both celecoxib and other NSAIDs, with celecoxib prescriptions showing a more pronounced rise over time. By the final interval (January–April 2024), celecoxib prescriptions surpassed those of other NSAIDs.

**Figure 2:**
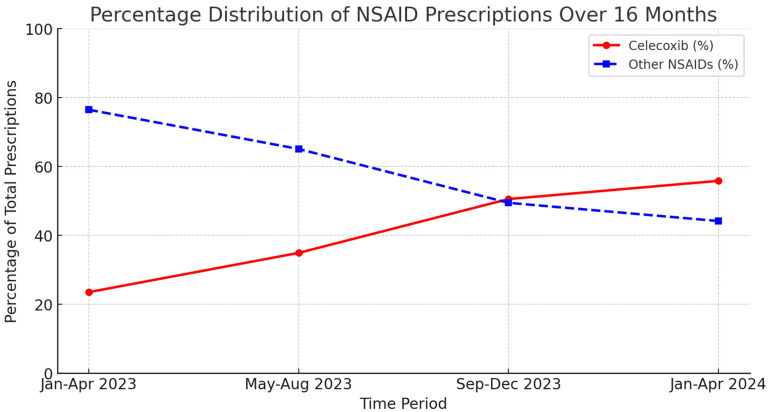
This line graph illustrates the percentage distribution of celecoxib (red) and other NSAIDs (blue) as a proportion of total NSAID prescriptions across four time periods. The data reveal a progressive increase in the percentage of celecoxib prescriptions with each interval. From September–December 2023, celecoxib use nearly equaled the combined prescriptions of all other NSAIDs. In the final period (January–April 2024), celecoxib prescriptions surpassed those of all other NSAIDs combined, suggesting that it became the preferred NSAID within the pain management clinic.

These findings are visually represented in Figure 1, which illustrates the total number of prescriptions for celecoxib and other NSAIDs over the 16-month study period, highlighting the increasing trend in celecoxib prescriptions compared to other NSAIDs. Additionally, Figure 2 presents the percentage distribution of celecoxib and other NSAID prescriptions over time, demonstrating the shifting proportion of celecoxib use relative to total NSAID prescriptions.

## 4. Discussion

Celecoxib was the first COX-2-selective inhibitor approved for use in OA and rheumatoid arthritis (RA) [16]. Multiple randomized double-blinded multicenter trials have demonstrated its superiority to placebos and non-inferiority to traditional, non-selective NSAIDs in improving the signs and symptoms of these conditions [15,18,19,20,21,28]. To clarify, all coxibs (including celecoxib) are a subclass of NSAIDs that selectively inhibit the COX-2 enzyme. In contrast, traditional NSAIDs such as diclofenac and ibuprofen are non-selective, meaning they inhibit both COX-1 and COX-2. This difference in enzyme selectivity helps explain variations in gastrointestinal and cardiovascular side effect profiles between drug classes. Since it was first introduced into pain management, evidence has also supported its use in chronic back pain, including two RCTs demonstrating its superior efficacy at treating chronic low back pain relative to the weak opioid agonist tramadol [29] and acetaminophen [17]. Its efficacy has also been demonstrated in the treatment of JRA, where at 3 and 6 mg/kg twice-daily doses, it is non-inferior to standard naproxen doses [22]. Additional evidence from the first decade of the century points to celecoxib’s efficacy in ankylosing spondylitis [30,31,32] and to a lesser extent in psoriatic arthritis, where a superiority to placebo was observed at two weeks but not at 12 weeks [33].

Beyond its clinical effectiveness, its pharmacokinetic properties play a crucial role in its suitability for long-term pain management. Celecoxib is a lipid-soluble, highly permeable drug that reaches peak plasma concentrations within two to four hours post administration, with an effective half-life of approximately 11 h [34]. It exhibits about 97% protein binding, primarily to albumin, undergoes hepatic metabolism via CYP2C9, and is excreted through feces and urine [35]. For chronic pain associated with OA, RA, or other inflammatory conditions like ankylosing spondylitis, the usual dosage is 200 to 400 mg/day, with the goal of using the lowest effective dose for the shortest amount of time. In a healthy adult receiving standard RA twice-daily doses, steady-state concentrations are reached within five days [35].

The mechanism of action of celecoxib is central to both its therapeutic effects and improved gastrointestinal safety profile relative to non-selective NSAIDs. Celecoxib binds to the sulfonamide side chain of the COX-2 enzyme, exerting reversible, time-dependent, and competitive inhibition by competing with arachidonic acid for access to the enzyme’s active site [36]. This blocks the conversion of arachidonic acid into prostaglandins, which mediate pain and inflammation [37]. Unlike non-selective NSAIDs, celecoxib selectively targets COX-2 by binding to a larger, hydrophobic side pocket that is absent in COX-1, allowing for sparing of COX-1. As COX-1 is constitutively expressed in gastric epithelial cells and plays a crucial role in synthesizing gastroprotective prostaglandins [38], its preservation reduces the risk of NSAID-associated gastrointestinal toxicity.

The safety profile of celecoxib has been extensively studied, particularly regarding its CV risks following the withdrawal of rofecoxib from the market due to results from the VIGOR trial reporting increased myocardial infarction and stroke risk with long-term use when compared to naproxen [39]. Early studies, such as a review of serious cardiovascular (CV) events among 2035 patients with a history of colorectal neoplasia enrolled in the Adenoma Prevention with Celecoxib trial, suggested a dose-dependent increase in the composite endpoint of CV death, myocardial infarction (MI), stroke, or heart failure when comparing celecoxib (200 or 400 mg twice daily) to a placebo [40]. However, these dosages and patient population were not representative of typical celecoxib use in chronic pain management. In contrast, the Celecoxib Long-term Arthritis Safety Study (CLASS) RCT exhibited a similar rate of adverse thromboembolic CV events with celecoxib at 400 mg twice daily compared with diclofenac [41]. More recent evidence, including the Prospective Randomized Evaluation of Celecoxib Integrated Safety versus Ibuprofen or Naproxen (PRECISION) trial, a large randomized controlled study of patients with high CV risk requiring NSAIDs for OA or RA, aligns with these findings by demonstrating that celecoxib was non-inferior to naproxen and ibuprofen in terms of CV safety as measured by a composite outcome of CV death, nonfatal MI, or nonfatal stroke [42]. Further supporting the non-inferiority of its CV safety profile in patients with RA and OA, a recent meta-analysis of 21 RCTs suggested that celecoxib does not increase the risk of CV events compared to non-selective NSAIDs or placebos [43].

Additionally, celecoxib maintains a favorable gastrointestinal and even renal safety profile compared to non-selective NSAIDs. In a secondary analysis of the PRECISION trial, celecoxib exhibited a more favorable GI side effect profile (including bleeding, obstruction, and symptomatic ulcers) than ibuprofen or naproxen when all these NSAIDs were co-administered with esomeprazole [44]. A different, more recent secondary analysis of the same trial found that celecoxib led to fewer renal side effects compared with ibuprofen or naproxen [45]. In line with these findings, the CLASS trial had previously shown that celecoxib, even at doses greater than those clinically indicated (twice the recommended dose for RA and four times the recommended dose for OA), was associated with lower incidence of symptomatic ulcers and their serious complications compared with ibuprofen and diclofenac at standard doses [46]. Similarly, other studies have shown that celecoxib was as effective as pelubiprofen in treating RA-related pain but with fewer gastrointestinal adverse effects [47] and led to lower rates of clinically significant upper or lower gastrointestinal events when compared to diclofenac co-prescribed with omeprazole in patients with OA and RA [48].

Despite celecoxib’s largely favorable safety profile compared to non-selective NSAIDs, NSAIDs as a class remain associated with both CV and gastrointestinal risks, necessitating careful consideration of patient selection. A large meta-analysis of individual participant data from randomized trials confirmed that while coxibs, including celecoxib, carry an increased risk of major vascular events compared to placebos, this risk is comparable to that of high-dose diclofenac and ibuprofen [49]. Additionally, the meta-analysis reinforced that all NSAID regimens, including coxibs, increase the risk of upper gastrointestinal complications [49], underscoring the importance of individualized risk assessment when selecting long-term NSAID therapy.

While these findings support the gastrointestinal and renal safety advantages of celecoxib, its broader clinical utility is particularly relevant in populations with complex pain management needs, such as older adults with multimorbidity. Chronic pain conditions such as OA, RA, and low back pain are more prevalent in aging populations [50,51,52]. Older adults also have a higher burden of chronic comorbidities, including CV disease, diabetes, and renal dysfunction [53,54], which complicate pain management by limiting medication options. Given this multimorbidity, coupled with age-related physiological declines in hepatic metabolism, renal clearance, and gastrointestinal function [55,56,57], pain medications for older patients should be suitable for long-term use, have a favorable safety profile, and minimize the risk of drug interactions and organ toxicity. In this context, celecoxib is a well-suited option for pain management in older adults, given its selective COX-2 inhibition, which reduces gastrointestinal toxicity compared to non-selective NSAIDs while providing effective analgesia. Its CV safety profile, demonstrated as non-inferior to ibuprofen and naproxen in high-risk populations, further supports its use in aging patients with multimorbidity. As the aging population continues to grow, optimizing pain management strategies while balancing efficacy and safety remains a critical priority, reinforcing the relevance of celecoxib as a viable alternative in long-term pain management.

Reflecting these considerations, prescribing patterns have begun to shift, with an increasing emphasis on non-opioid analgesics like celecoxib in contemporary pain management. The rising trend in celecoxib prescribing within our institution reflects these broader shifts in pain management practices. Similarly, a retrospective observational analysis using Medical Expenditure Panel Survey data found a nationwide increase in non-opioid pain medication prescriptions among U.S. adults with moderate to severe pain from 2014–2018, reinforcing this shift toward opioid-sparing strategies [58]. This trend highlights the growing emphasis on evidence-based pain management strategies. Celecoxib’s clinical efficacy, combined with its relatively safer gastrointestinal side effect profile and CV tolerability being non-inferior to traditional alternatives, makes it an appealing option for long-term pain management. As efforts to reduce opioid dependency continue, celecoxib presents a promising alternative for patients requiring effective and safer long-term treatment options.

Opioids have been widely prescribed for chronic pain, with over 20% of adults experiencing chronic pain using a prescription opioid as of 2019 [59]. Opioid use increases with age for the treatment of moderate to severe chronic pain, peaking among adults aged 45 to 64 [59]. However, chronic opioid use is associated with significant adverse effects, including cognitive impairment, immune suppression, and gastrointestinal dysfunction [60]. Opioid use has also been linked to increased pain sensitivity, mood disorders, and a heightened risk of substance use disorder and dependency [61,62,63], with older adults particularly vulnerable to these complications [59].

Over the past decade, prescription opioid use has contributed to more than 14,000 deaths annually [64]. In response to the opioid epidemic, clinicians are increasingly encouraged to prioritize non-opioid treatments for managing chronic pain. The Center for Disease Control and Prevention (CDC) released the Guideline for Prescribing Opioids for Chronic Pain in 2016 to officially recommend prioritizing non-opioid analgesics and stricter opioid prescribing practices [65]. Implementation of these guidelines, along with state-level regulations such as prescription limits and “pill mill” laws—legislation designed to target clinics that prescribe opioids without legitimate medical justification—was estimated, as of 2017, to be associated with an 11.4% reduction in the proportion of patients with a history of long-term opioid use who continued opioid therapy, and an 11.7% increase in adults with chronic pain initiating treatment with non-opioid analgesics instead of opioids [66]. Additionally, among patients with chronic pain and a history of long-term opioid use, guideline implementation was linked to a 131.4% increase in the use of non-opioid-only analgesic regimens [66].

As the shift toward opioid-sparing pain management strategies continues, non-selective NSAIDs and COX-2 selective inhibitors, including celecoxib, have become key components of multimodal analgesic approaches [67]. While opioids may provide superior short-term analgesia in certain cases, NSAIDs offer both analgesic and anti-inflammatory effects, making them effective for conditions such as OA, RA, and other chronic pain syndromes. Importantly, unlike opioids, NSAIDs do not carry the risks of respiratory depression, sedation, constipation, or cognitive impairment, which can be particularly detrimental in older adults. Furthermore, they do not induce physiological dependence or addiction, addressing one of the primary concerns associated with long-term opioid use.

The observed increase in celecoxib prescribing in our patient population over a relatively short 16-month period may reflect heightened clinician awareness of its favorable gastrointestinal and cardiovascular safety profile, particularly in patients with multiple comorbidities. This trend may also align with institutional efforts to reduce opioid prescribing and emphasize non-opioid analgesics in chronic pain management. While our study did not collect pathology-specific data, future investigations may benefit from exploring diagnostic correlations to better understand prescribing preferences.

### Implications for Clinical Practice

Over a 16-month study period, we observed an eight-fold increase in the prescription of celecoxib by board-certified pain management physicians practicing in a single specialized clinic. The increasing reliance on celecoxib within this pain management clinic underscores its growing importance as a treatment option for chronic pain. While COX-2 selective inhibitors, particularly celecoxib, offer efficacy with improved gastrointestinal safety compared to traditional, non-selective NSAIDs, their appropriate use remains critical to minimizing risks and optimizing patient outcomes.

NSAIDs, as a class, contribute to 30% of hospital admissions for preventable medication-related adverse events [68], yet many physicians remain unfamiliar with the full spectrum of NSAID-associated risks and the guidelines for their use in specific patient populations [69]. This knowledge gap may extend to patients, who can often be ill-informed about potential adverse events, leading some to self-escalate doses in attempts to enhance pain relief [70]. Moreover, NSAID use is frequently underreported in patient records, either because of underreporting by patients or suboptimal documentation by providers [71], further complicating risk assessment and management.

Evidence-based decision making is therefore essential to ensure a safe and effective integration of celecoxib into mainstream pain management strategies. Clinicians should carefully consider absolute and relative contraindications, including hypersensitivity to NSAIDs, aspirin-sensitive asthma, pregnancy, severe heart failure, active GI bleeding, inflammatory bowel disease, cerebrovascular bleeding, and significant hepatic or renal impairment. While celecoxib exhibits a more favorable gastrointestinal profile, prescribers must remain vigilant regarding increased overall risk for CV and gastrointestinal adverse effects after celecoxib initiation, as well as long-term risks associated with long-term use of any NSAID, such as renal and hepatic toxicity [72,73], particularly in vulnerable populations.

Additionally, proper patient education is crucial. Patients should be counseled on the importance of adhering to prescribed doses and tactfully made aware of potential side effects, including CV risks and renal impairment, to prevent misuse and improve overall safety. As the use of opioid-sparing strategies continues to expand, ensuring that both physicians and patients are well-informed about the risks and benefits of celecoxib is paramount for maximizing its therapeutic value in chronic pain management.

## 5. Limitations

This study has limitations which may affect the interpretation and generalizability of the findings. First, this study was conducted within a single pain management clinic, limiting the generalizability of its findings. While the results provide valuable insights into the prescribing patterns of celecoxib and other NSAIDs in this specific setting, they may not reflect trends in other clinics or healthcare systems with different patient demographics, resources, or prescribing practices. Variations in prescribing practices, physician training, and formulary availability could result in different trends in NSAID utilization across institutions.

Additionally, although the 16-month study period offered valuable insights into prescribing trends, it represents a relatively brief timeframe for assessing long-term changes in clinical practice. As a result, it remains uncertain whether the observed increase in celecoxib prescriptions signifies a temporary fluctuation influenced by specific events, such as educational initiatives, or a more enduring shift in prescribing behavior. Expanding the study period to multiple years would provide a more comprehensive evaluation, enabling a clearer understanding of the trends’ sustainability and the factors driving these changes over time.

Despite these limitations, this study provides valuable preliminary insights into the changing trends in NSAID prescribing within a pain management clinic. Future research addressing these limitations, including larger-scale, multicenter studies with extended follow-up periods and detailed patient-level data, will help to develop a more comprehensive understanding of the role of celecoxib and other NSAIDs in chronic pain management.

## 6. Conclusions

This study highlights a significant upward trend in celecoxib prescribing within a single pain management clinic over a 16-month period. The findings underscore the growing importance of selective COX-2 inhibitors in chronic pain management and suggest a shift in prescribing practices that may be influenced by a combination of patient needs, clinical guidelines, and physician preferences. Further research is needed to explore the factors underlying these trends and their implications for patient care.

## Data Availability

The original contributions presented in this study are included in the article. Further inquiries can be directed to the corresponding author.
